# Afghan medical students’ perceptions, and experiences of their medical education and their professional intentions: a cross-sectional study

**DOI:** 10.1186/s12909-023-04577-7

**Published:** 2023-08-11

**Authors:** Muhammad Haroon Stanikzai, Mohammad Hashim Wafa, Khalid Akbari, Zabihullah Anwary, Ahmad Haroon Baray, Hadia Sayam, Abdul Wahed Wasiq

**Affiliations:** 1 Public Health Department, Faculty of Medicine, Kandahar University, Kandahar, Afghanistan; 2 Neuropsychiatric and Behavioral Science Department, Faculty of Medicine, Kandahar University, Kandahar, Afghanistan; 3 Internal Medicine Department, Faculty of Medicine, Paktia University, Paktia, Afghanistan; 4 Clinic Department, Faculty of Medicine, Bost University, Helmand, Afghanistan; 5 Para-Clinic Department, Faculty of Medicine, Malalay Institute of Higher Education, Kandahar, Afghanistan; 6 Internal Medicine Department, Faculty of Medicine, Kandahar University, Kandahar, Afghanistan

**Keywords:** Afghanistan, Medical students, Medical education, Conflict, Career choices

## Abstract

**Background:**

Numerous challenges have crippled the Afghan healthcare system on individual, organizational, and societal levels. The Afghans have acknowledged that an evidence-based perspective is paramount to enhancing medical training capacities across the country, which may, in turn, best ensure appointing highly competent authorities to address health system problems on such multiple levels.

**Objectives:**

This study assessed current Afghan senior medical students’ perceptions, and experiences of their medical education and their future professional intentions.

**Methods:**

We conducted this cross-sectional study at seven public and private Afghan medical institutes from March to April 2022. We invited 665 senior medical students through an anonymous survey using the Google survey online forms via social-media platforms, such as WhatsApp Messenger. Descriptive statistics were employed for the data analyses.

**Results:**

The mean age (± SD) of the students was 23.7 (± 2.2) years and males constituted 79.9% (510) of the study sample. About 22.6% of them rated their medical training as excellent, and nearly a third of them (37%) said that it is good. Nearly half (48.7%) of the students would prefer to stay in Afghanistan. The leading motives for moving overseas were to obtain more advanced and quality education (69.9%), and a decent personal life (43.9%). Nearly two-thirds (67.4%) of them asserted that current political and armed conflicts in Afghanistan may have influenced their professional choices.

**Conclusion:**

This study epitomizes that the quality of medical education in Afghanistan has room for growth and development to meet the standards set on regional and global grounds.

**Supplementary Information:**

The online version contains supplementary material available at 10.1186/s12909-023-04577-7.

## Introduction

Afghan universities have always been centers for enticing political turmoil because Afghan higher education, in general, and medical education, in particular, have not received the attention it calls for throughout Afghan history [1].

Afghans established modern higher education institutions after 1950. Soviet military invasion and the ensuing civil war have seriously disrupted the developmental process of these institutions, and due to the destabilizing process, that engulfed the entire Afghan society, the Afghan medical system was profoundly affected [2].

The infiltrators made universities nonfunctional for a long time, and they killed or obliged numerous health professionals to emigrate. Hospitals, medical schools, and their professional personnel were targets to demoralize the community and undermine the ruling system. The ensuing civil war precipitated female deprivation of any professional training, which resulted in a grave deficiency of female health professionals. The latter has been a hampering problem in Afghan society, where female patient examination by a male doctor is cultural misconduct [3].

Moreover, numerous challenges had a crippling effect on the Afghan healthcare system. For instance, in addition to the health workforce challenges, the current national data inform us of a shortage of proper planning and policy development at the country level [4, 5]. The Afghans have acknowledged that the need for an evidence-based perspective is paramount to enhancing medical training capacities across the country. It may, in turn, best enable us to address health-related problems on individual, organizational, and community levels [6].

In the last two decades, however, with the assistance of the international society through implementing copious innovative and quality-enhancing projects, Afghans have somewhat managed to erect, equip and develop numerous facilities for Afghan healthcare and medical education. We reopened university doors to female students and updated the teaching methods. The international assistance led us to rebuild the infrastructure and develop a contemporary unified medical curriculum for Afghan medical education. Medical education flourished and thrived. To epitomize this claim, we reconditioned former medical schools and established new medical education institutes throughout the country [1, 2, 4].

In terms of enhancement of medical training capacities, currently, more than 40 public and private medical schools are providing theory and practicum to medical students across the country [1, 2]. Annually, these institutes graduate approximately 4000–5000 medical graduates. Additionally, as an initiative of the Ministry of public health, implemented by the Afghanistan Medical Council (AMC), every public or private medical school graduate must pass the exit exam to register as a medical practitioner. Moreover, 10–15% of the graduates have recruited annually through merit-based exams for the Master of Public Health (MPH) and clinical specialization programs held in the medical faculties of national universities and secondary care facilities of public hospitals, respectively [5, 6]. Despite these untiring efforts toward a quality health system and clinical excellence, there is an enormous room for capacity building, quality enhancement, and financial independence of the organization, to name a few. Consequently, numerous medical students graduate from these programs with insufficient medical competencies and low professional motivation.

The nearly half-a-century-long war that continues had profound effects on all aspects of Afghan lives, leaving medical education with no exception. Even though a few public and private medical schools cater to improving medical education, Afghan medical education is prone to many overt and covert sinister factors [7, 8]. Overt factors are lack of proper infrastructure, financial restraints, and loss of well-experienced and knowledgeable faculty members [7]. The underhanded or psychological factors consist primarily of an under-reflection of, for instance, a healthy attitude towards studentship and professionalism, empathy among the medical students, and being hopeful for the light at the end of the tunnel [2, 7]. Briefly, the educational and even the basic human needs of these elite youths who have dedicated a vital chunk of their lives to the welfare of the broader society have been prone to neglect in this war-torn country. Thus, we aimed to assess the perceptions, experiences, and future professional intentions of currently enrolled Afghan senior medical students. Such information could guide better decision-making in policy advocacy and planning for competent human resources in a country torn by decades of conflict and engulfed by, at least, an undermined healthcare system. Additionally, our findings will probably contribute insight to improving the delivery model and overall quality of medical education in Afghanistan.

## Materials and methods

### Study design and study setting

We conducted this cross-sectional study at seven public and private Afghan medical institutes from March to April 2022. Kabul Medical University (KMU) in Kabul province, Aryana Medical University (AMU) in Jalalabad province, Bost University in Helmand province, Kandahar University (KDRU) in Kandahar province, Paktia University in Paktia province, National (Milli) University in Kabul province, and Malay Institute of Higher Education (MIHE) in Kandahar province were the seven study sites for our data collection. They were selected through convenience sampling.

### Inclusion and exclusion criteria

We included medical students who had completed at least two years of their medical education. Owing to the essence of our research parameters, especially perceptions, experiences, and professional intentions, which are probably well-defined in senior students, we declined to include first and second-year students. Medical students who were absent at the time of the study and those who refused to participate were also excluded.

Our recruiters did not incline toward general attributes of the students, such as residential area (rural/urban), religious affiliations, political orientations, minority status, absence of physical disability, or their socio-economic background. These factors may somehow discount the reflection of studentship in our findings.

### Sample size calculation and sampling methods

The estimated sample size was 665, obtained by a sample size calculation based on the official records of the Afghan Ministry of Higher Education (MoHE). We have documented the number of medical students with a 95% confidence level, a 5% margin of error, a design effect of 1.5, and a 10% non-response rate. The Afghan medical students’ perceptions and experiences of medical education and their professional intentions were unknown; therefore, a maximum variation of 50% was assumed.

The sample population was distributed among the seven institutes using a systematic sampling method. The study participants were selected using a simple random sampling technique (MS Excel, 2019).

### Study measures and data collection

We developed the questionnaire through an exhaustive and careful observation of the pertinent literature [see Additional file 1]. We did not hesitate to employ the experiences of Afghan professors with medical education, perceptions of medical students, and their professional intentions in the questionnaire development process. Additionally, we checked the pertinence and conformity of the questionnaire with the previous work that assessed the perceptions, experiences, and professional intentions of medical students in other conflict-affected countries [9].

The questionnaire consisted of four parts. The first section included questions about participants’ socio-demographic characteristics (11 questions). The second section focused on the quality of their current medical education (5 questions). The third section asked about perceptions and their experiences of medical education and their future career plans (16 questions). Finally, the effects of conflict and insecurity on professional choices were asked (6 questions). Questions related to the quality of medical education had four options (Excellent, Good, Fair, and Poor). Participants’ perceptions and experiences of medical education were assessed using a 5-point Likert scale. For each statement, respondents were asked to state their level of agreement as do not agree, mostly disagree, mostly agree, strongly agree, and not sure.

We translated the English version of the questionnaire into local languages (Pashtu and Dari). Then, the principal investigators and language experts reviewed the Pashtu and Dari translations. Eventually, they agreed upon a consensus translation. Prior to the commencement of the study, the questionnaire was pre-tested in a pilot study to check and revise (if required) its verbal consistency and structural reliability. The internal consistency (Cronbach’s alpha) value for the local version in the pre-tested sample was 0.926.

The recruitment strategy involved a staged process that included gathering WhatsApp registered numbers and email addresses of the randomly selected students, followed by the electronic distribution of the Pashtu and Dari versions of the questionnaire through WhatsApp Messenger and email to the chosen participants. The online form of the questionnaire was available for responses from March 10, 2022, to April 25, 2022. A consent form appeared on the first page of the questionnaire depicting the study description, objectives, and participants' right to withdraw at any time they wish. If the medical student would like to participate, they could willfully provide their consent and choose their preferred language for the form. Only then did the participants proceed to complete and submit the anonymous survey questionnaire. This approach allowed for the easy and efficient distribution of the study tool, which was crucial in ensuring high participation rates and accurate data collection.

### Statistical analysis

The data were transferred from Microsoft Excel 2019 to IBM SPSS Statistics version 21.00, where it was cleaned and analyzed [10]. We employed descriptive statistics to understand medical students' perceptions, experiences, and professional intentions regarding medical institutions throughout Afghanistan. The results are presented in figures and tables as numbers (percentages) and means (standard deviation [SD]).

## Results

From the seven medical institutes, a total of 638 (response rate, 95.9%) students individually completed the questionnaire. More than half (375; 58.8%) of them were enrolled in public institutes, and the rest (263; 41.2%) were studying in private institutes. The mean age of the students was 23.7 (± 2.2 SD) years with a range of 20–32 years, and nearly two-thirds (418; 65.5%) of them were younger than 25 years. Most of the participants were male (510, 79.9%), and a majority (283, 60%) of them were single. Two-thirds of the students reported a relative who was a medical doctor, and only 9.5% reported having a doctor relative who is living abroad. Table 1 depicts the baseline characteristics of these medical students.

**Table 1:** Baseline characteristics of the medical students (*n* = 638)

Variables	Frequency (%)
Age (years)	
1. 20–22	228 (35.7)
2. 23–24	190 (29.8)
3. 25–32	220 (34.5)
Sex	
1. Male	510 (79.9)
2. Female	128 (20.1)
Living arrangement	
1. Home	348 (54.5)
2. Dorm	290 (45.5)
Marital status	
1. Single	283 (60)
2. Married	255 (40)
Type of medical school	
1. Public	375 (58.8)
2. Private	263 (41.2)
Name of the medical institute	
1. Kabul Medical University	141 (22.1)
2. Kandahar University	123 (19.3)
3. Paktia University	111 (17.4)
4. Boost University	73 (11.4)
5. Aryana (Nangrahar) University	65 (10.2)
6. National (Milli) University	64 (10.0)
7. Malalay Institute of Higher Education	61 (9.6)
Year in medical school	
1. Third	195 (30.6)
2. Fourth	200 (31.3)
3. Fifth or sixth	243 (38.1)
Academic performances	
1. Top 1/3	210 (32.9)
2. Middle 1/3	408 (63.9)
3. Bottom 1/3	20 (3.1)
He/She has family members who live abroad	
1. Yes	195 (30.6)
2. No	443 (69.4)
He/She has a relative who is a doctor	
1. Yes	378 (59.2)
2. No	260 (40.8)
He/She has a relative who is a doctor and lives abroad (*n* = 195)	
1. Yes	80 (41)
2. No	115 (59)

The medical students were not very fond of the quality of their medical education; about 22.6% (25.3% public vs. 18.6% private) rated their medical training as excellent, and nearly a third of them (37%, 32.5% public vs. 43.3% private) said that it is good (Table 2). The strongest positive opinions about their medical training were related to the management of their clinical rotations. A fourth of the students (161; 25.2%) reported that their clinical rotations were well organized. Another 15.2% of the students reported that the basic sciences curriculum was well organized and handled. Some 13.8% of them said that the faculty tries to keep up with contemporary medical developments on a global scale. The strongest negative opinions were about how the scheduled sessions are organized (42.9%), how dedicated the faculty is to helping students learn (32.6), and to access the textbooks and journals (28.1%). Table 2 portrays the detailed notion of the students about their medical training.

**Table 2:** Perceptions of Afghan medical students regarding the aspects of their medical training (*n* = 638)

	**Strongly** **disagre** **N (%)**	**Somewhat** **disagree** **N (%)**	**Somewhat** **agree** **N (%)**	**Strongly** **agree** **N (%)**	**Not** **sure** **N (%)**
Clinical rotations are well organized	106 (16.6)	170 (26.6)	124 (19.4)	161 (25.2)	77 (12.2)
We have access to updated textbooks and journals	179 (28.1)	210 (32.9)	103 (16.1)	91 (14.3)	55 (8.6)
Sessions are conducted as scheduled	274 (42.9)	236 (37.0)	97 (15.2)	22 (3.4)	9 (1.4)
Instructors keep up to date	151 (23.7)	231 (36.2)	149 (23.3)	88 (13.8)	19 (3.0)
Instructors are generally motivated and competent teachers	208 (32.6)	249 (39.0)	133 (20.8)	35 (5.5)	13 (2.1)
Instructors’ private business do not interfere with teaching responsibilities	146 (22.9)	156 (24.5)	128 (20.1)	129 (20.2)	79(12.4)
The basic science curricula are well organized	156 (24.5)	242 (37.9)	116 (18.2)	97 (15.2)	27 (4.2)
		**Poor**	**Fair**	**Good**	**Excellent**
**Overall perceived quality of medical training**		52 (8.2)	206 (32.2)	236 (37)	144 (22.6)
Perceived quality of medical training (Public)		38 (10.1)	120 (32.0)	122 (32.5)	95 (25.3)
Perceived quality of medical training (Private)		14 (5.3)	86 (32.6)	114 (43.3)	49 (18.6)

**Fig. 1:**
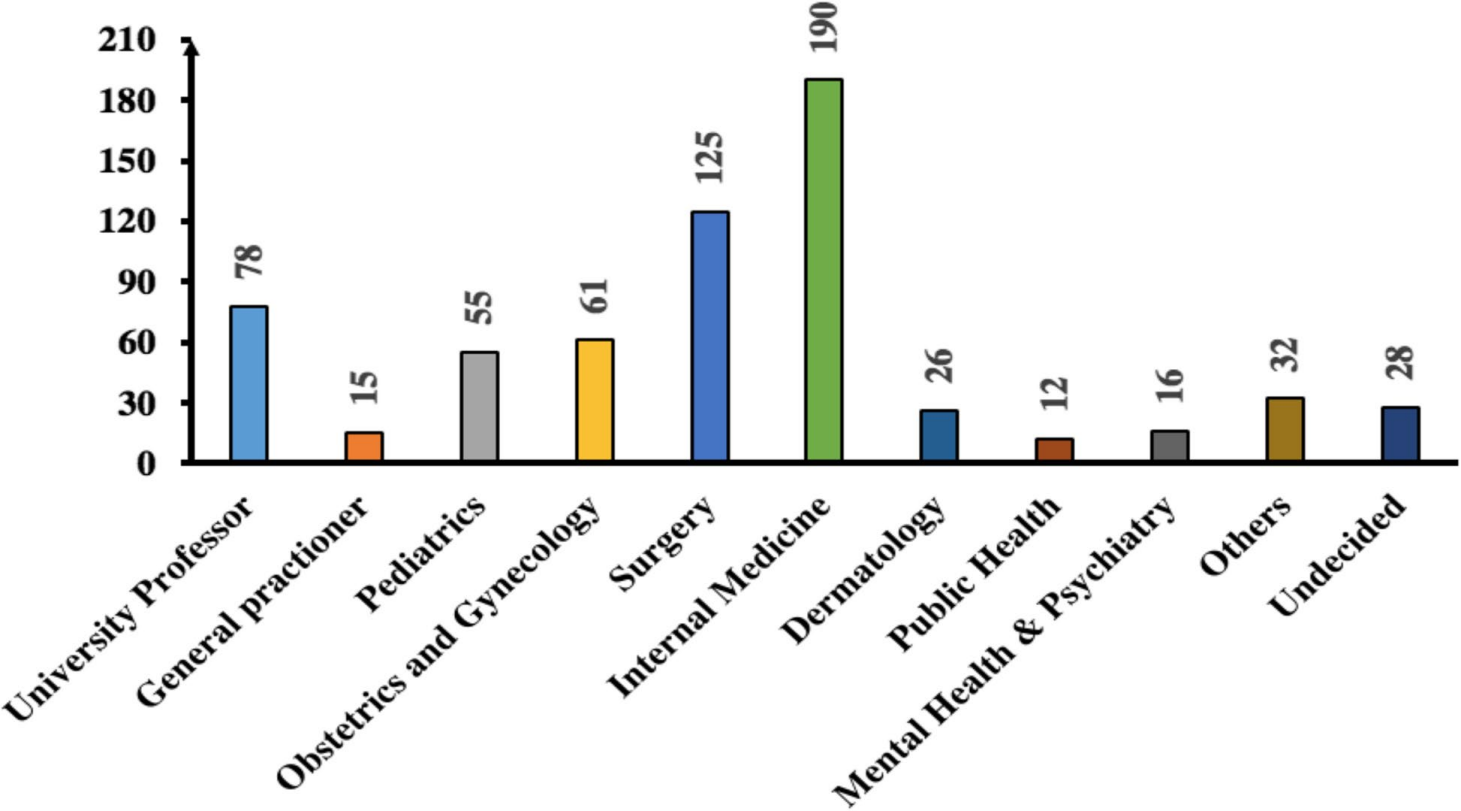
Professional intentions of the surveyed Afghan medical students

Almost all the medical students (95%) said that they were starting to envision their clinical specialty preferences (Fig. 1). Of them, more than a third (245, 38.1%) said that they would preferably pursue their post-graduate training in Afghanistan. Of those (393, 61.9%) who indicated that they would pursue their postgraduate training overseas, only 181 (28.4%) had picked a specific venue. Overall, internal medicine was the most envisioned postgraduate program (190, 29.7%), followed by 125 (19.6%) students inclined to specialize in surgery. Other choices included being a university professor (78, 12.2%), an OB–GYN specialist (61, 9.5%), a pediatrician (55; 8.6%), and a dermatologist (26; 4%). Only 12 (1.8%) of them had a proclivity to dig deeper into public health.

Table 3 describes the motives of the students for either staying in Afghanistan or moving overseas, after graduation. Nearly half (48.7%, 311) of the students said that they would prefer to stay in Afghanistan. About 18.4% (117) of the students said that they considered traveling overseas either only for obtaining quality training and returning back to their homeland, or staying overseas permanently. Another third (32.9%, 210) of the students said that they have occasionally thought of moving overseas. Their leading reasons to stay in Afghanistan were a feeling of responsibility to the motherland and assisting the afflicted Afghans (77.1%, 492), being with family and friends (50.5%, 322), and being familiar with the Afghan health care system (43.1%). In contrast, the leading motives for moving overseas were to obtain more advanced education (69.9%, 446), highly professional training (46.7%, 298), and an anticipation of a decent personal life (43.9%, 280) (Table 3).

**Table 3:** Motivation for staying or traveling overseas (*n* = 638)

	**Most important N (%)**	**Somewhat important N (%)**	**Not very important N (%)**	**Least important N (%)**
**Motivation for staying in Afghanistan**				
To be with family and friends	322 (50.5)	166 (26.0)	41 (6.4)	109 (17.1)
Familiar with health care system	275 (43.1)	209 (32.7)	49 (7.7)	105 (16.5)
Feel responsibility	492 (77.2)	60 (9.4)	36 (5.6)	50 (7.8)
I can get a better position/job	245 (38.4)	161 (25.2)	82 (12.9)	150 (23.5)
Personal lifestyle	280 (43.9)	128 (20.1)	64 (10.0)	166 (26.0)
**Motivation for traveling overseas**				
Better personal life	186 (29.2)	116 (18.2)	185 (29.0)	151 (28.6)
Avoid war and conflict	295 (46.2)	108 (16.9)	147 (23.0)	88 (13.8)
Further studies	446 (69.9)	61 (9.6)	85 (13.3)	46 (7.2)
Better professional opportunities	298 (46.7)	140 (21.9)	149 (23.4)	51 (8.0)
Better pay and working conditions	159 (24.9)	120 (18.8)	218 (34.2)	141 (22.1)
To be with family	174 (27.3)	120 (18.8)	283 (44.4)	61 (9.6)
	**Not at all**	**Occasionally**	**Frequently**	**All the time**
Thinking of traveling overseas	311 (48.7)	210 (32.9)	45 (7.1)	72 (11.3)

Nearly two-thirds (67.4%, 430) of the students said that political and armed conflict in Afghanistan influenced their professional choices. Nearly one in four students (159, 24.9%) asserted losing at least one member of the nuclear family to armed conflict since 2000. There were 126 (19.7%) students who reported the death of a classmate or a medical faculty due to armed conflict. Nearly half (44.5%, 284) of the students reported that at least one of their faculty members had moved overseas (Table 4).

**Table 4:** The effects of conflicts and insecurity on the professional choices of Afghan medical students (*n* = 638)

Variables	Frequency (%)
The extent of career choice affected	
1. Very much	430 (67.4)
2. Somewhat	169 (26.5)
3. A little bit	24 (3.8)
4. Not at all	15 (2.4)
A nuclear family member killed	
1. Yes	159 (24.9)
2. No	479 (75.1)
A classmate or a faculty member died in the war	
1. Yes	126 (19.7)
2. No	512 (80.3)
A family member moved overseas	
1. Yes	284 (44.5)
2. No	354 (55.5)

## Discussion

To the best of our knowledge, this is the first study that assesses the professional intentions of Afghan medical students as well as their perceptions and experiences related to the medical education they are attending. The results of this study will most probably contribute to improving the delivery model and overall quality of medical education in Afghanistan.

The majority of students (59.6%) in this study reported that the quality of the medical education they received was either excellent or good. However, 8.2% expressed their concern about the quality of medical education. Parallel studies conducted on medical students in diverse developing countries show mixed results on the quality of the tendered medical education [9, 11–13]. We argue that students' judgment may lack a standardized model of comparison. From a more realistic standpoint, however, an insufficiency of proper infrastructure, improper management, financial restraints, and ever-increasing loss of well-experienced and knowledgeable faculty members are hypothesized to be the crucial underlying factors that may have led to a lower quality of medical education in Afghanistan [2, 5, 6]. Policymakers, authorities at the MoHE, and other stakeholders involved in Afghan medical education provision should acknowledge these facts and that the students are increasingly interested and entitled to better medical education. Thus, the officials at the MoHE level and the level of medical institutes should actively partake in designing, documenting, implementing, and supervising quality medical education, with an additional emphasis on managing basic sciences curricula and providing the system and the students with contemporary educational materials.

Most participants (61.9%) indicated they intend to pursue postgraduate training overseas. This finding may have presumably resulted from some factors. Firstly, overseas is an enormous platform that offers the student immensely diverse postgraduate programs or super-specialties. Secondly, the medical marketplace, be it a university or a hospital, favors the resume of professionals with a degree from overseas. Thirdly, studying overseas, especially in the context of a scholarship, usually offers a unique opportunity for study. Students studying in their homeland are obliged to super add to their load of professional studies, shouldering their families, and realizing their social responsibilities, which are highly time-consuming and distracting. Finally, leaving your comfort zone and living in a socio-culturally different environment is a pretty enriching and globally favored life experience. Studies have shown that postgraduate training enrollment influences students' decisions concerning permanent residency in their host country [14–19]. Internal medicine and surgery were typical career choices reported by medical students. The prevalent and ever-growing popularity of these specialties may have a potential role in the preferences of the surveyed students [20, 21]. Despite, and probably because of the over-40-year-long history of almost perpetual crises that have engulfed the entire country, especially the health sector and its workforce, Afghanistan had not yet developed a robust management plan for, at least, health-related human resources at a national level [2, 4, 7]. The findings of this study suggest that policymakers should prioritize and allocate extra resources to quality postgraduate training across the country and develop motivational and support strategies for enticing medical graduates to pursue diverse specialties and subspecialties.

Our findings reveal that the emigration intentions of Afghan medical students (51.3%) correspond to those of medical students from other developing countries [14, 17, 19]. Advanced and quality medical education and yearning for a decent personal life were the most frequent motives influencing the respondent's emigration intentions. Furthermore, nearly half (44.5%) of the students reported that at least one of their faculty members had moved overseas. Studies with analog findings show that postgraduate training and career opportunities, perceived higher level of income, along with favorable working and living conditions influence opting for career paths of both medical students and qualified physicians [14–19].

To contextualize, Afghanistan has experienced over-four-decade of unprecedented conflict, violence, and insecurity on multiple fronts. The ongoing multidimensional conflict and its subsequent venomous insecurity have undoubtedly impaired medical education on several levels [5, 22]. We observed that about 67.4% of the students asserted that political and armed conflict in Afghanistan may have influenced their professional choices. Studies from other conflict-ridden countries have documented additional psychological stress among students and a lower quality of teaching [9, 17]. Of course, multidimensional support from local, regional, and international providers is of paramount significance to improving the quality of medical education in Afghanistan, which will, in turn, invariably assure the establishment of quality health services in the future.

## Limitations

Not unexpectedly, our findings have limitations, primarily related to the data collected in major Afghan cities. The perceptions, experiences, and professional intentions of Afghan medical students who study in socioeconomically and geopolitically minor cities may somehow differ from the collected data. Secondly, female students (20.1%) and students with low academic performance (3.1%) were under-represented in our sample. Thirdly, we have not assessed the bio-psycho-social health of our subjects, their current consumption of any psychoactive medication, and their socio-political affiliations that may have somehow confounded their uttered responses.

## Conclusion

The findings from this study provide insights into the perceptions, experiences, and professional intentions of medical students who currently study in major Afghan cities. This study reveals that the quality of medical education in Afghanistan has room for growth and development to meet the standards set on regional and global grounds. Therefore, we consider the following points worthwhile recommendations to the Ministry of higher education of Afghanistan. Firstly, the ministry has to acknowledge that the national medical education policy calls for an exhaustive revision, in which issues pertinent to sex, skill mix, and misdistribution have to be particularly addressed. Secondly, the ministry is to improve the quality of public and private medical education through designing and implementing advanced and internationally approved training programs for the capacity building of the academic staff and the employment of full-time qualified educators. Thirdly, the ministry is to introduce mandatory exit exams at all graduate levels, and integrate medical entrance exams with the national entrance exam (Kankor) to reduce potential corruption. Fourthly, teaching methods are to be updated and altered to problem-based and case-based learning. Besides, access to the latest versions of reputed medical literature has to be facilitated. The ministry is to supervise and monitor academic and administrative activities at all levels of the public and private medical schools through adopting innovative regulatory mechanisms.

### Supplementary Information

**Additional file 1.**

## Data Availability

The primary data used to support the findings of this study are available with the corresponding author upon request.

## Declarations

**Ethics approval and consent to participate**

The Research and Ethics Committee of Kandahar University approved the study (Certificate #88). All methods were carried out in accordance with the World Medical Association (WMA)’s Declaration of Helsinki. We discussed the nature and objectives of the study with the Ministry of Higher Education (MoHE) officials and obtained their permission. We received an electronic version of the informed consent from all participants.
