# Supplementary material for: Afghan medical students’ perceptions, and experiences of their medical education and their professional intentions: a cross-sectional study
Source: BMC Med Educ. 2023 Aug 11;23:569. doi: 10.1186/s12909-023-04577-7 (PMC10416376; doi:10.1186/s12909-023-04577-7)
Supplement: Supplementary file 1 — Additional file 1. [file 12909_2023_4577_MOESM1_ESM.pdf]

Afghan medical students' perceptions, and experiences of their medical education and their professional intentions: A cross-sectional study

Running title: The perceptions, experiences and professional intentions of Afghan Medical students

Authors

[1] Muhammad Haroon Stanikzai, MD, MPH (haroonstanikzai1@gmail.com)

[2] Mohammad Hashim-Wafa, MD, Ph.D (wafahashim@gmail.com)

[3] Khalid Akbari, MD, MPH (khalidakbari486@gmail.com)

[4] Zabihullah Anwary, MD, MPH (zabihullah_anwary@yahoo.com)

[1] Ahmad Haroon Baray, MD, MPH (ahmadharoon.baray@gmail.com)

[5] Hadia Sayam, MD (hadiasayam7@gmail.com)

[6] Abdul Wahed Wasiq, MD (waheed330@gmail.com)

Affiliations

[1] Public Health Department, Faculty of Medicine, Kandahar University, Kandahar, Afghanistan

[2] Neuropsychiatric and Behavioral science Department, Faculty of Medicine, Kandahar University, Kandahar, Afghanistan

[3] Internal Medicine Department, Faculty of Medicine, Paktia University, Paktia, Afghanistan

[4] Clinic Department, Faculty of Medicine, Bost University, Helmand, Afghanistan

[5] Para-clinic Department, Faculty of Medicine, Malalay Institute of Higher Education, Kandahar, Afghanistan

[6] Internal Medicine Department, Faculty of Medicine, Kandahar University, Kandahar, Afghanistan

Corresponding author:

Name: Dr. Muhammad Haroon Stanikzai, MD, MPH (JHSPH)

Email: haroonstanikzai1@gmail.com

Address: Medical Faculty, Near Ayno Mena, 10th District, Kandahar, Afghanistan

Afghan curative Medical Student Questionnaire perception, expectation future plans

A. About yourself

No	Variables	Responses
1	Age	
2	Your sex	1. Male 2. Female
3	Marital status	1. Single 2. Married
4	Permanent residential address	
5	Living arrangement	1. Home 2. Dorm
6	Name of your medical school	
7	Type of medical school	1. Government 2. Private
8	Your year in medical school (Circle)	3 4 5 6
9	Your overall ranking in class	1. Top % 2. Middle 3. Bottom
10	Do you have a relative who is a doctor?	1. Yes<br>2. No
11	Do you have a brother, sister, or a parent living outside of Afghanistan now?	1. Yes<br>2. No<br>If No Skip Q12 and Q13
12	Where (list all locations)?	
13	Are any of these relatives outside of Afghanistan a doctor or a dentist?	1. Yes<br>2. No

B. The quality of your medical education

14. How would you rate the overall quality of your medical training at this school? *(circle only one)*

1 Excellent 2 Good 3 Fair 4 Poor

B.1 How would you rate each of these aspects of your medical training at this school? (check each line)

**1**=do not agree **2**=mostly disagree **3**=mostly agree **4**=strongly agree 5=not sure						
		1	2	3	4	5
15	Faculty knowledge and dedication to teaching is very high					
16	Faculty always show up to conduct teaching sessions when scheduled					
17	Faculty private practice responsibilities interferes with teaching responsibilities					
18	Faculty keep up to date in the latest developments in their field					
19	Faculty are generally good teachers with a strong interest in helping us learn					
20	The basic sciences curricula were generally well organized to help me learn					
21	Clinical rotations are well generally organized to help me learn the topic thoroughly					
22	Access to current textbooks and journals is generally good					

23. How would you compare the quality of your medical training in Afghanistan to what you know of medical schools in other countries in the region? *(check only one)*

1 Much better	*2 About the same*	*3 Worse*	*4 Much worse*	*5 Do not know*

24. What areas of your medical education do you think were particularly good?

25. What of your medical education do you think were particularly poor?

26. Do you have any suggestions to improve medical education in Afghanistan?

C. Future plans

27. Which of the following statements best describes your future educational plans? ***Circle only one***

1	I am still undecided about my future plans
2	On graduation I will not seek further training, and become a general practitioner
3	Family Medicine
4	Anesthesia
5	Pediatrics
6	OBS/GYN
7	Surgery or the surgical specialties (ENT, urology, neurosurgery etc)
8	Internal medicine or the medical specialties (cardiology, nephrology, etc)
9	Radiology
10	Dermatology
11	Public Health or Community Medicine
12	Pathology
13	Psychiatry
14	Other (specify)____

28. Which statement best describes your plans at this time for further training after graduation?

1	I have no plans at this time
2	I am trying to decide, and have some idea
3	I have clear ideas about what I will do on qualification

29. If you have some plans (b-c) even if not completed, where will you do this training?

1	I will definitely do my specialty train in Afghanistan
2	I am actively looking to do my specialty training outside Afghanistan, but no specific plans
3	I have definitely and specific plans to do my specialty training outside Afghanistan

C.2 Where do you see yourself working after you complete all of your training?

	**1** = definitely not **2** = possibly **3** = very likely	**1**	**2**	**3**
30	Central provinces			

31	Afghanistan, but not central provinces			
32	Other Middle East countries including the Persian Gulf			
33	Europe			
34	Australia or New Zealand			
35	Canada			
36	United States			

37. How often do you think of leaving Afghanistan after your graduation from medical school? *(circle one)*

1 Not at all	2 Occasionally	3 Frequently	4 All the time

C.3 What would be your biggest motivators for living in Afghanistan after you complete all of training?

1 = most important 2 = somewhat important 3 = not very important 4 = least important		**1**	**2**	**3**	**4**
38	I am familiar with the health care system in Afghanistan				
39	To be with my family and friends				
40	This is my country and I feel responsibilities				
41	The positions I can get here are better than I can get outside Afghanistan				
42	The personal life style in Afghanistan is what I like				

C.4 How would you rate your motivators for going outside of Afghanistan?

**1** = most important **2** = somewhat important **3** = not very important 4 = least important		1	2	3	4
43	Seek advanced training				
44	Better professional opportunities				
45	Be with family				
46	Better pay and working conditions				
47	Avoid war and conflict				
48	Better personal lifestyle				

49. In the past 3 years do you think the desirability to practice medicine as a young doctor in Afghanistan has-- *(circle one)*

1 Gotten better	2 stayed the same	3 Gotten worse	4 not sure

50. Do you think the professional satisfaction that doctors get from the practice of medicine in Afghanistan has--

1 Gotten better	2 stayed the same	3 Gotten worse	4 not sure

51. In the past 3 years to you think the potential of living a satisfying personal life while practicing medicine in Afghanistan has— *(circle one)*

1 Gotten better	2 stayed the same	3 Gotten worse	4 not sure

D. In the next section are some questions about your opinions of health care in Afghanistan now

D.1 how would you rate health care in Afghanistan today?

	**1** = Excellent **2** = Good **3** = Fair 4 = Poor 5= Don't know	1	2	3	4	5
52	The quality of health care received in hospitals					
53	The quality of health care received in Primary Health Care Clinics (PHCCs)					
54	The quality of health care received in private clinics					
55	The availability of medicines and supplies when needed					
56	The availability of laboratory tests when needed					
57	The attitude and concern of doctors workers for patients					
58	The attitude and concern of other health care workers for patients					
59	The attitude of patients and their family toward doctors					
60	Salary and income for doctors					
61	Working conditions for doctors					
62	Safety and security for doctors					

D.1 How would you rate the availability of the following components of health care in urban areas?

	**1** = More than enough **2** = Adequate **3** = Shortage 4 = Severe shortage	1	2	3	4
63	The number of public hospitals				
64	The number of Primary Health Care Clinics (PHCCs)				
65	The number of private clinics				
66	The number of nurses in hospitals and clinics				
67	The number of technicians (laboratory, x-ray, pharmacy etc)				
68	The availability of medicines and supplies when needed				
69	The availability of laboratory tests when needed				

E. The effects of conflict and insecurity on your professional choices

70. To what extent are your career choices affected by the political and conflicts in Afghanistan *(circle one)*

1 Very much 2 Somewhat 3 A little bit 4 Not at all

71. Did any immediate family (parents or siblings) members die in the war that started in 2000?

1 Yes 2 No

A. If so, how many? _______

B. How many immediate family members were seriously injured? _______

72. Did any of your medical school classmates or faculty die in the war that started in 2000? Y N

A. If so, how many? _______

73. Do you know of any medical school faculty members who left Afghanistan during your medical school years? *1 Yes 2 No*

If so, how many do you know of? ______
